# Prognostic Value of Combined Radiomic Features from Follow-Up DWI and T2-FLAIR in Acute Ischemic Stroke

**DOI:** 10.3390/jcdd9120468

**Published:** 2022-12-19

**Authors:** Alessia Gerbasi, Praneeta Konduri, Manon Tolhuisen, Fabiano Cavalcante, Leon Rinkel, Manon Kappelhof, Lennard Wolff, Jonathan M. Coutinho, Bart J. Emmer, Vincent Costalat, Caroline Arquizan, Jeannette Hofmeijer, Maarten Uyttenboogaart, Wim van Zwam, Yvo Roos, Silvana Quaglini, Riccardo Bellazzi, Charles Majoie, Henk Marquering

**Affiliations:** 1 Department of Electrical, Computer and Biomedical Engineering, University of Pavia, 27100 PV Pavia, Italy; 2 Department of Biomedical Engineering and Physics, Amsterdam UMC Location University of Amsterdam, 1105 AZ Amsterdam, The Netherlands; 3 Department of Radiology and Nuclear Medicine, Amsterdam UMC Location University of Amsterdam, 1105 AZ Amsterdam, The Netherlands; 4 Department of Neurology, Amsterdam UMC Location University of Amsterdam, 1105 AZ Amsterdam, The Netherlands; 5 Department of Radiology and Nuclear Medicine, Erasmus MC University Medical Center, 3015 Rotterdam, The Netherlands; 6 Department of Neuroradiology, Centre Hospitalier Universitaire de Montpellier, 34400 Montpellier, France; 7 Department of Neurology, Centre Hospitalier Universitaire de Montpellier, 34400 Montpellier, France; 8 Department of Neurology, Rijnstate Hospital, 6836 BH Arnhem, The Netherlands; 9 Department of Neurology and Department of Medical Imaging Center, University Medical Center Groningen, 9713 GZ Groningen, The Netherlands; 10 Department of Radiology and Nuclear Medicine, Maastricht University Medical Center, 6229 HX Maastricht, The Netherlands

**Keywords:** acute ischemic stroke, functional independence, imaging bio-markers, machine learning, ensemble tree classifier, explainable AI, SHAP

## Abstract

The biological pathways involved in lesion formation after an acute ischemic stroke (AIS) are poorly understood. Despite successful reperfusion treatment, up to two thirds of patients with large vessel occlusion remain functionally dependent. Imaging characteristics extracted from DWI and T2-FLAIR follow-up MR sequences could aid in providing a better understanding of the lesion constituents. We built a fully automated pipeline based on a tree ensemble machine learning model to predict poor long-term functional outcome in patients from the MR CLEAN-NO IV trial. Several feature sets were compared, considering only imaging, only clinical, or both types of features. Nested cross-validation with grid search and a feature selection procedure based on SHapley Additive exPlanations (SHAP) was used to train and validate the models. Considering features from both imaging modalities in combination with clinical characteristics led to the best prognostic model (AUC = 0.85, 95%CI [0.81, 0.89]). Moreover, SHAP values showed that imaging features from both sequences have a relevant impact on the final classification, with texture heterogeneity being the most predictive imaging biomarker. This study suggests the prognostic value of both DWI and T2-FLAIR follow-up sequences for AIS patients. If combined with clinical characteristics, they could lead to better understanding of lesion pathophysiology and improved long-term functional outcome prediction.

## 1. Introduction

Stroke is the second leading cause of death worldwide, with ischemic stroke being the most frequent type [1]. Acute ischemic stroke (AIS) is caused by thrombotic or embolic occlusion of a cerebral artery leading to reduced blood supply to part of the brain and consequent cell death. Treatments aim for the timely restoration of the blood supply to the occluded artery. The standard of care involves administering thrombolytic drugs (intravenous thrombolysis) followed by mechanical retrieval of the clot (endovascular treatment). Currently, the choice of the best therapy is the subject of ongoing research. Moreover, ischemic lesion evolution after stroke is a complex biological process. Notably, a considerable proportion of patients do not experience clinical improvement despite successful recanalization of the occluded artery and reperfusion of the ischemic area [2].

Although several well-known risk factors, such as lifestyle, common comorbidities, and aging, have been already identified as related to stroke onset and severity, several challenges remain open in this field, including the prediction of long-term prognosis and the identification of biomarkers for common treatment complications, to name only a few.

Neuroimaging has significantly improved AIS management, with magnetic resonance imaging (MRI) and computed tomography (CT) being the preferred imaging modalities. The use of MRI is particularly associated with a significant decrease in the rates of complications in AIS patients at the expense of only marginally increased length and cost of hospitalization [3]. Moreover, MRI is the most sensitive noninvasive neuroimaging technique for assessing brain changes after stroke as well as in predicting recovery, especially in the subacute stage [4]. Accurate functional outcome prediction after treatment is needed in order to provide patients with a prognosis, plan rehabilitation programs, and provide research avenues targeting secondary treatments [5,6].

Previous studies exploring the prognostic value of MRI features have identified the size of the area with diffusion restriction and the apparent diffusion coefficient (ADC) as being associated with clinical outcome. Furthermore, infarct volume measurements (in the subacute phase between 14 h and 48 h) are predictors of long-term functional outcome, measured in terms of 3-month modified Rankin Scale (mRS) [7,8,9]. A texture analysis based on ADC maps and T2-FLAIR images highlighted an association between image texture features and functional outcome [10].

It is not always easy to fully explain the reasons for poor functional outcome and to timely predict its occurrence in AIS patients. In this context, the predictive power of machine learning (ML) and deep learning (DL) models and their ability to extract hidden patterns from neuroimages have been explored in several studies [11,12]. Most of the published works focused on pre-treatment imaging features extracted from a single imaging modality. However, a tool able to support the interpretation and prognostic value assessment of follow-up neuroimaging sequences in the subacute stage would be useful in clinical practice. There could be early detectable signs for unfavorable long-term clinical outcome among follow-up imaging characteristics that could help to gain new insights into the lesion evolution mechanism and its pathophysiology. MRI follow-up protocols usually include DWI and T2-FLAIR sequences to assess ischemic lesion status after treatment. DWI is considered the gold standard modality for evaluating the status of the lesion in the subacute stage thanks to its ability to quantify the movement of water molecules [13]. During ischemia, intracellular water accumulates in the injured cells as a consequence of changes in the osmotic gradients caused by the malfunctioning of the sodium–potassium pump. This results in a bright signal in MR diffusion imaging, allowing the detection of infarction and cytotoxic edema. On the other hand, T2-FLAIR is a structural imaging sequence in which T2 prolongation is commonly observed hours after stroke. T2 prolongation is mainly related to increased water content in the ischemic tissue, and usually represents vasogenic edema [14]. Although several prognostic models have been proposed in the literature [15,16], lack of reproducibility and interpretability of the prediction results is often the most critical aspect. Moreover, most of the related works are single-center or single-country studies, and thus present a significant risk of bias. Finally, none of the previous studies have explored the prognostic value of combined imaging features extracted from these two follow-up MR sequences; yet, these may actually capture different aspects of stroke lesion status after treatment, and multimodal predictive modeling approaches could lead to more accurate and reliable prognosis prediction from a clinical perspective.

The aim of this study is to evaluate the prognostic value of the combination of two follow-up MR sequences. We hypothesize that the prediction of stroke functional outcome benefits from combined follow-up DWI and T2-FLAIR imaging features in terms of 90-day mRS. We aim to build a complete prognostic model considering both follow-up imaging features extracted from the two sequences and routinely available clinical characteristics, thereby exploiting all patient data available up to 24 h. Finally, we are interested in exploring the contribution of each feature to the prediction of the prognostic model and studying the clinical interpretation of the identified imaging predictors.

## 2. Materials and Methods

**Figure 1:**
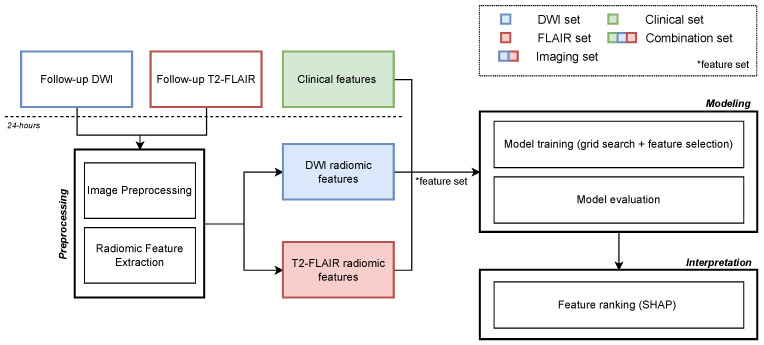
Schematic representation of the study workflow. Follow-up DWI and T2-FLAIR scans are preprocessed and radiomic features are extracted from the segmented lesions. Five different sets of features are then used to train an ensemble tree-based ML classifier to predict binary 90-day mRS (0–2 vs. 3–6). Each set respectively includes: (1) only DWI radiomic features (*DWI set*), (2) only T2-FLAIR radiomic features (*FLAIR set*), (3) both DWI and T2-FLAIR radiomic features (*imaging set*), (4) only clinical characteristics (*clinical set*), and (5) a combination of all the radiomic features and the clinical characteristics available up to 24 h (*combination set*). Finally, the models’ interpretability is evaluated using SHapley Additive exPlanations (SHAP).

The complete study workflow is schematized in Figure 1. We propose an image analysis pipeline, starting with the preprocessing of the MR scans and the infarct lesion segmentation in DWI and T2-FLAIR sequences. Radiomic features are subsequently extracted from the segmented lesions and combined with clinical characteristics in order to train and validate an ensemble tree-based ML classifier for outcome prediction. Finally, the models’ interpretability is evaluated using SHapley Additive exPlanations (SHAP) [17].

### 2.1. Data Collection

The patients included in this study were enrolled in the MR CLEAN-NO IV [18] trial, which is a multicenter randomized clinical trial including 539 patients ≥18 years of age with a pre-stroke mRS <3 with an intracranial large vessel occlusion (LVO) confirmed on CT/MR angiography within 4.5 h of symptom onset eligible for both intravenous thrombolysis (IVT) and endovascular treatment (EVT). The patients were randomized to receive IVT followed by EVT or EVT only. The follow-up imaging protocol included NCCT or MRI at 24 h for all patients. A detailed description of the inclusion and exclusion criteria is provided in the study protocol [19]. The trial was carried out in 20 hospitals in the Netherlands, Belgium, and France. Therefore, the slice thickness of the scans varied (from 3 to 6 mm), and the field strength used for the acquisition was 1.5 or 3 Tesla.

Baseline clinical features, 24-h NIH Stroke Scale score, and 90-day mRS were available for all the patients included in the study.

**Figure 2:**
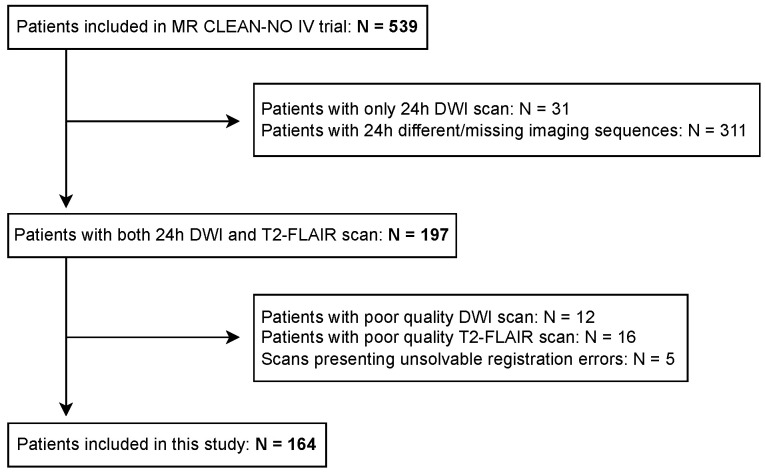
Flow chart of the patient inclusion process.

Figure 2 shows a flow-chart of the patient inclusion process. We included patients with high-quality follow-up DWI and T2-FLAIR scans, resulting in 164 patients from MR CLEAN-NO IV trial. Scans presenting severe artifacts were excluded from this study.

### 2.2. Image Preprocessing

**Figure 3:**
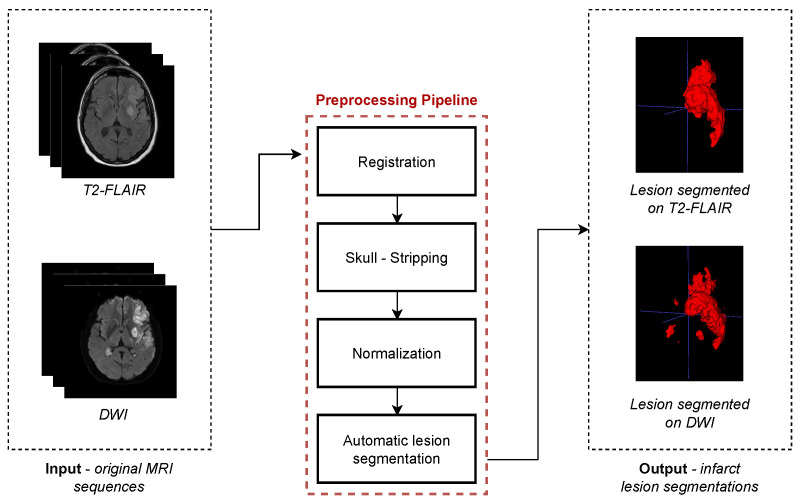
Image preprocessing pipeline. The same steps are repeated for each of the input MR sequences, obtaining the infarct lesion segmentation as the final output from both imaging sequences.

The main image preprocessing steps are summarized in Figure 3. All the scans were skull-stripped and registered to the standard MNI-space via rigid transformations using the open source SPM12 toolbox [20], obtaining an isotropic voxel dimension of 1 mm. Intensities were normalized using the white stripe normalization method [21]. Finally, the lesion was segmented in both imaging sequences.

**Figure 4:**
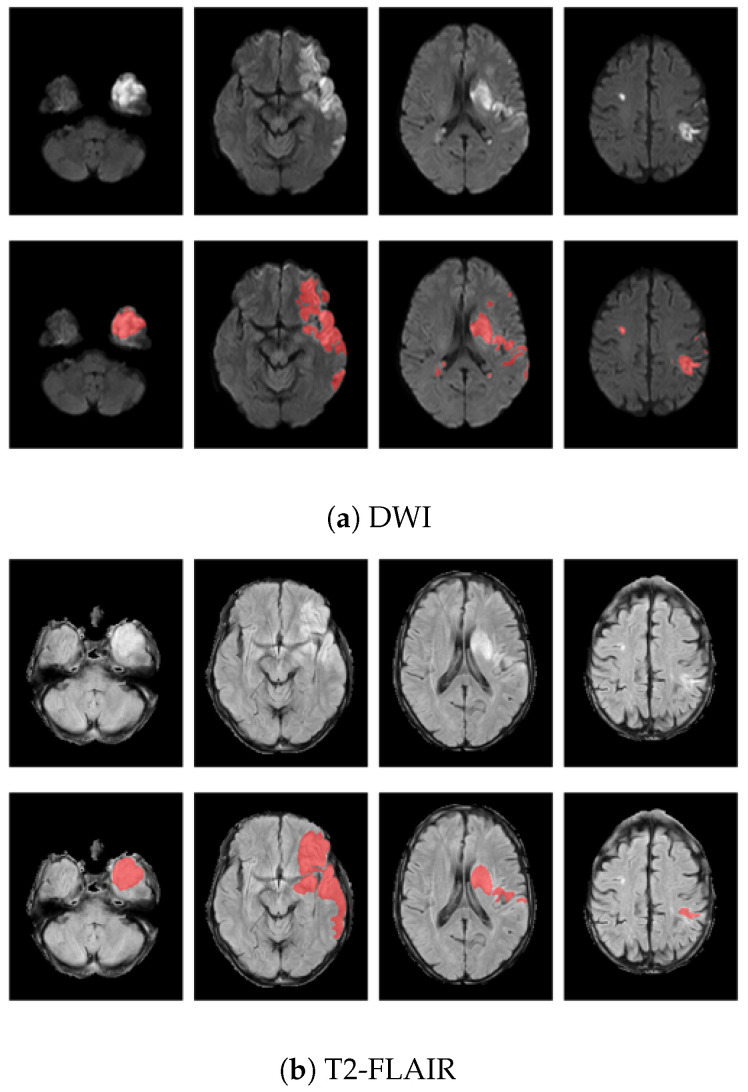
Example of infarct lesion segmentation on a selected slices sampled from (**a**) DWI and (**b**) T2-FLAIR preprocessed volumes. For each modality, original slices are displayed in the first row and lesion segmentation (red) is shown as overlapping the correspondent slices in the second row.

Infarcts in DWI scans were automatically segmented using a deep learning software developed in-house and then manually checked and corrected by two expert neuroradiologists (with more than twenty and five years of experience, respectively) using ITK-SNAP software [22]. Infarcts in T2-FLAIR sequences were automatically segmented using DeepNeuroSeg [23], a deep fully convolutional network trained to detect white matter hyperintensities (WMH) from FLAIR that ranked first in WMH Segmentation Challenge at MICCAI 2017. All resulting segmentations were manually checked and corrected using ITK-SNAP software. Figure 4 shows an example of T2-FLAIR and DWI lesion segmentation from the same patient. For each modality, a few slices sampled from the preprocessed scan are reported in the first line. Lesions appear hyperintense on both imaging modalities, and the segmented area after the preprocessing steps (red) is displayed as overlapping the original slices in the second row.

### 2.3. Features

Radiomic features were extracted from the segmented lesion, which is defined as the region of interest (ROI) in the following using the open-source PyRadiomics [24] Python package. Although deep neural networks have been proven to be powerful feature extractors in several computer vision applications, and are able to capture hidden patterns in images, we decided to avoid this strategy in our study due to the limited sample size and consequent high risk of overfitting. The extracted radiomic features (n=107) extensively describe the lesion, capturing different aspects, and can be subdivided in the following classes: first-order statistics, size/shape based, and high-order textural characteristics. The first-order statistics describe the distribution of voxel intensities within the ROI (e.g., energy, entropy); the size- and shape-based features include both 2D and 3D measures, among which is the follow-up lesion volume, which is often used in literature as a prognostic marker; finally, the textural features include measures derived from correlation matrices describing spatial patterns of voxel intensities. There are five main textural matrices: (1) the Gray Level Co-occurrence Matrix (GLCM), which describes the second-order joint probability function of an image region constrained by the mask; (2) the Gray Level Size Zone Matrix (GLSZM), which quantifies gray level zones in an image, defined as a the number of connected voxels that share the same gray level intensity; (3) the Gray Level Run Length Matrix (GLRLM), which quantifies gray level runs, defined as the length in number of pixels, of consecutive pixels that have the same gray level value; (4) the Neighbouring Gray Tone Difference Matrix (NGTDM), which quantifies the difference between a gray value and the average gray value of its neighbors within a defined distance; and (5) the Gray Level Dependence Matrix (GLDM), which quantifies gray level dependencies in an image, defined as a the number of connected voxels within a defined distance that are dependent on the center voxel.

We considered five different sets of features to predict binary 90-day mRS (0–2 vs. 3–6) as the model outcome, a clinical definition for favorable long-term functional independence. Each set respectively includes: (1) only DWI radiomic features (named the *DWI set*, n=107); (2) only T2-FLAIR radiomic features (named the *FLAIR set*, n=107); (3) both DWI and T2-FLAIR radiomic features (named the *imaging set*, n=214); (4) only clinical characteristics (named the *clinical set*, n=23); and (5) a combination of all the radiomic features and the clinical characteristics available up to 24 h (named the *combination set*, n=237).

Clinical characteristics were included in the final models, as it is well known that a number of these are strongly associated with functional outcome. Therefore, we decided to further evaluate the prognostic value of imaging features on top of routinely acquired clinical characteristics in order to simulate a complete model exploiting all the patient data available up to 24 h. Clinical characteristics included: clinical history data (e.g., age, gender), baseline clinical measurements and imaging derived scores routinely acquired in the acute phase (e.g., glucose, ASPECTS), treatment, and post-treatment characteristics (e.g., type of treatment, 24-h NIH Stroke Scale). A complete list of the clinical characteristics considered in this study is provided in the Supplementary Materials.

### 2.4. Image Analysis Pipeline and Experimental Setup

We adopted the Extreme Gradient Boosting (XGB) model as a classifier, implemented in the XGBoost Python library [25]. XGB is a high-performing ensemble tree-based algorithm designed to deal with high dimensional and mixed input data types. One of the strengths of this algorithm is its capability to automatically handle collinear features and missing data; therefore, we preferred to avoid any imputing strategy for the missing clinical values.

We implemented a feature selection strategy based on SHAP values [17]. Lundberg et al. [26] showed that several common methods for estimating feature importance are inconsistent, as they can lower a feature’s assigned importance when the true impact of that feature actually increases. Therefore, they developed TreeSHAP, a consistent individualized feature attribution algorithm for tree ensembles based on SHAP values. It has been shown that this method results in reduced computational complexity depending on the maximum depth of the trees instead of the number of possible feature combinations, and it has demonstrated obtained results in better agreement with human intuition.

**Figure 5:**
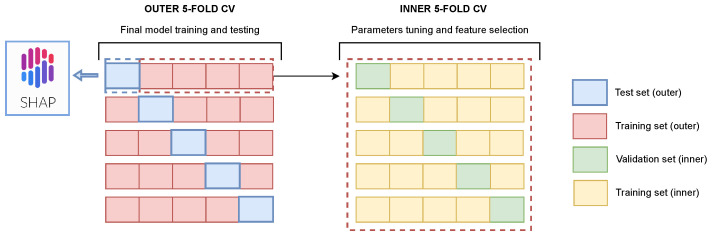
Schema of experimental setup, showing a schematic representation of the nested cross validation in the outer loop on the left and the inner loop on the right. Each row on the left represents the whole dataset, which is divided in five different folds; one fold (blue) is used as an independent test set, while the remaining four folds form the training set. Repeating this split five times ensures that each single fold is considered as a test set once. In the inner loop on the right, the training set is subsequently split in five folds again in order to obtain a validation set (green) used to estimate the model’s performance during the grid search and feature selection procedure. The best model with the chosen parameters and features is then retrained on the outer training set (red) and tested on the outer test set (blue). SHAP values for the final model interpretability are computed on the test sets.

Because the analyzed sample size is relatively small, nested stratified *k*-fold cross validation (CV) was used to train and test the model in order to avoid biases and overly-optimistic results. The schema of the experimental setup is shown in Figure 5, where the outer and inner loops are schematically represented on the left and right, respectively. In our pipeline, *k* was set to 5, meaning that that 20% of the dataset is used as a test set at each split. In the inner CV, a grid-search strategy was implemented to tune the model’s parameters (learning_rate, max_depth, min_child_weight, colsample_bytree, colsample_bynode, subsample, alpha) in order to maximise validation accuracy. A detailed description of the parameters is available in the official documentation [27]. For each feature, mean absolute SHAP values were computed on each validation set of the inner loop. The average SHAP values at the end of inner CV were used to obtain the final feature importance ranking. All the features with a mean absolute SHAP value equal to 0 where automatically discarded. For each round of the outer CV, the final model with the selected parameters and features was retrained on the whole outer training set and tested on the unseen test set to assess the model’s generalization capabilities.

The pipeline was executed for each of the five feature sets previously defined. For each classifier, final average performance on the test sets was evaluated in terms of classification accuracy, precision, sensitivity, specificity, and area under the ROC curve (AUC). We compared the models pairwise based on their AUC using DeLong’s test, with the highest AUC as a reference.

### 2.5. Explainability

The explainability of AI models is of the utmost importance in the medical field, especially when dealing with challenging clinical tasks such as prognosis prediction where a black-box approach would be useless in clinical practice. The final aim in this case is not only to build a reliable model, it is to better understand the impact of the identified predictors that could possibly help to gain new insights into lesion pathophysiology and help clinicians to interpret and trust the model’s outcome.

For the best model, SHAP values were computed on the test sets to better understand the role of each feature in predicting the desired outcome. We used SHAP summary plots to show the most important features with the largest impact on outcome prediction for the best experiment. This is an easy to read beeswarm plot colored by feature values, with the features ordered based on their impact on the model’s output. Positive SHAP values are associated with unfavorable outcomes, while negative values are associated with good clinical outcomes. In order to visually inspect the meaning of the extracted radiomic characteristics, we ordered our cohort of patients based on the relevant features shown by the resulting SHAP summary plot and analyzed four different case studies.

## 3. Results

A complete list of the clinical characteristics, including clinical history and measurements, baseline imaging scores, and treatment and post-treatment characteristics of the patients included in this study and the original MR CLEAN-NO IV population, is provided in the Supplementary Materials.

**Figure 6:**
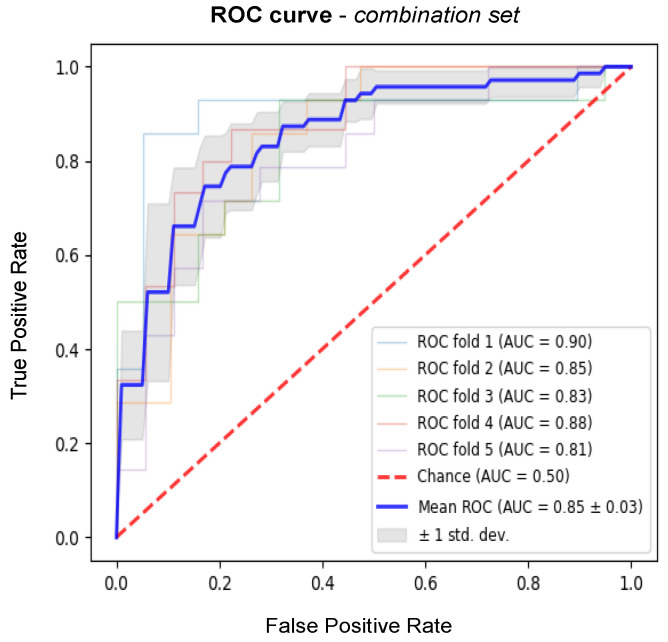
Average ROC curve (blue) obtained from the outer cross-validation of the best-performing model (*combination set*).

The prediction accuracy measures computed on the test sets for each collection of features are reported in Table 1. Table 2 provides the results of DeLong’s test. The model including all imaging features (*imaging set*) shows slightly higher performance compared to the models using features from a single modality. The average AUC achieved with the *imaging set* is 71% (+2% and +3% compared to the average AUC of *DWI* and *FLAIR set*, respectively). The model trained on the *clinical set* shows a higher predictive power compared to models using imaging features alone (+12% compared to the average AUC of the *imaging set*). Finally, the *combination set* led to the best prognostic model, outperforming all the other models in terms of the considered metrics, with an average AUC of 85% (Figure 6).

**Figure 7:**
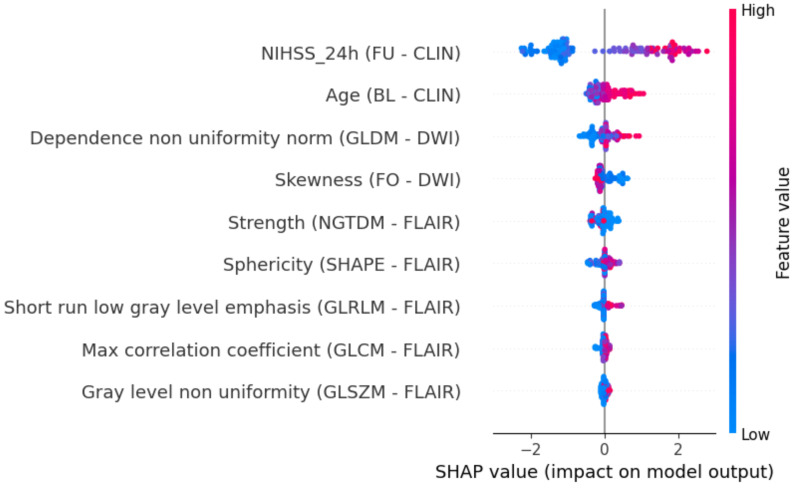
SHAP value beeswarm plot of the most relevant predictors on the test sets. Features are sorted according to their impact on model output. Positive and negative SHAP values are associated with unfavorable and favorable clinical outcomes, respectively. The colors represent the original feature values, with higher values in pink and lower values in blue. For each clinical feature (CLIN), the time of measurement is reported (BL = baseline, FU = follow-up at 24 h). For each radiomic feature, the imaging modality (FLAIR or DWI) and the type of feature are provided in brackets (FO = first order, SHAPE = 2D or 3D shape, NGTDM/GLRLM/GLCM/GLSZM = texture).

Figure 7 shows the SHAP summary plot of the most important features with the largest impact on outcome prediction for the best experiment (*combination set*). The features with the largest impact include both clinical characteristics and radiomic features. It can be seen that both DWI and T2-FLAIR radiomic features are ranked among the most relevant predictors, with texture features the most frequent ones. The only exceptions are *Skewness* measured on DWI, which is a first-order feature and an indicator of asymmetry in gray level distribution, and *Sphericity* measured on T2-FLAIR, which is a shape-related feature measuring the roundness of the shape of the lesion region. All the other image predictors are texture features, and despite the different textural matrices being able to capture slightly different aspects of gray values distributions, they are essentially describing the heterogeneity of the examined region (a detailed description of each feature is reported in the official documentation [28]). The beeswarm plot shows that more heterogeneous lesions (↑ Dependence non uniformity, GLDM–DWI; ↓ Strength, NGTDM–FLAIR; ↑ Short run low level emphasis, GLRLM–FLAIR; ↑ Max correlation coefficient, GLCM–FLAIR; ↑ Gray level non uniformity, GLSZM–FLAIR) are more associated with unfavorable clinical outcomes.

**Figure 8:**
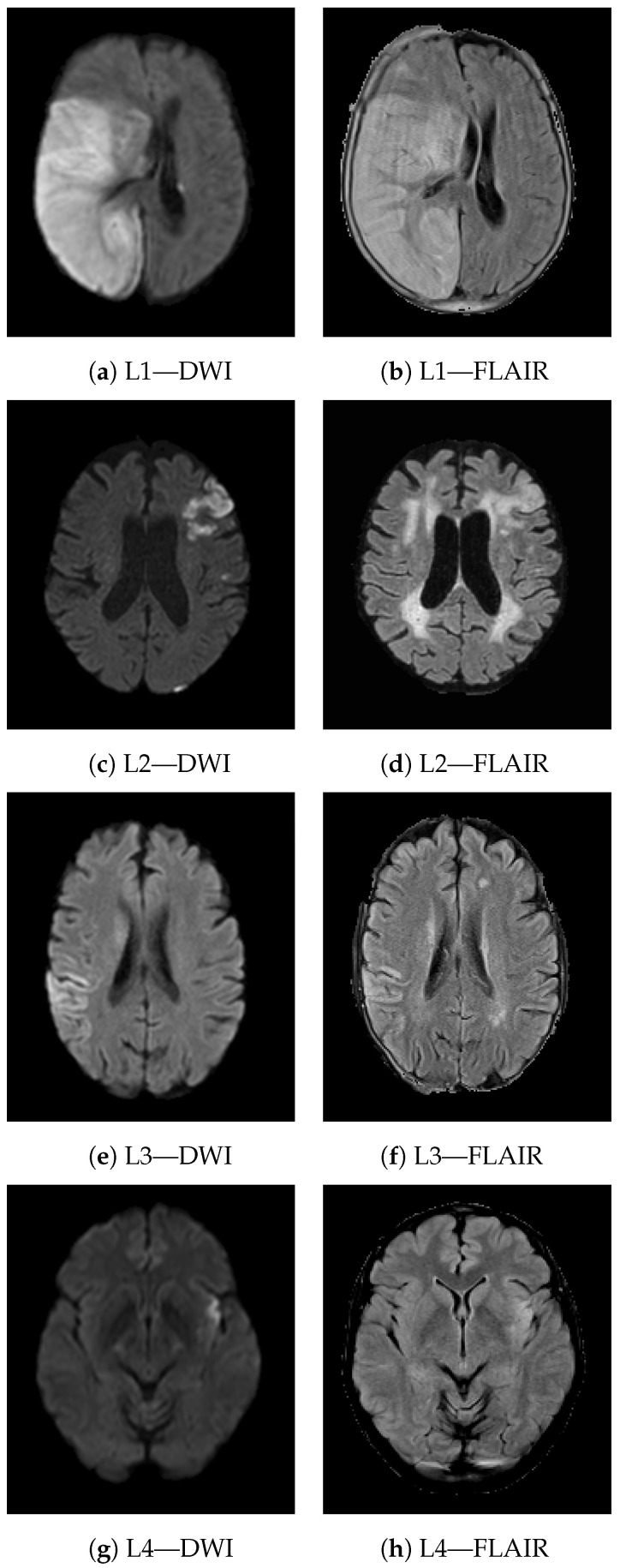
Axial views of DWI and T2-FLAIR scans from four different lesions (L1–L4). The lesions look hyperintense on both imaging sequences. L1 and L2 are two examples of patients with poor long-term functional outcome (90-day mRS of 6 and 5 respectively). L1 is quite extensive and presents PH1 hemorrage. Both lesions include WM and GM areas of the brain (texture heterogeneity measured in terms of Dependence non uniformity normalized—GLDM on DWI of 0.23 and 0.13, respectively). L3 and L4 instead are two examples of patients with good long-term functional outcome (90-day mRS of 2 and 0, respectively). Both lesions involve GM only (texture heterogeneity measured in terms of Dependence non uniformity normalized - GLDM on DWI of 0.08 and 0.04, respectively).

Figure 8 shows four different study cases (L1-4) demonstrating the meaning of texture heterogeneity for visual inspection. For each lesion, the axial view from both DWI and T2-FLAIR modality is reported. The lesion appears hyperintense on both imaging sequences. L1 and L2 are two examples of lesions from patients experiencing poor long-term functional outcome (90-day mRS of 6 and 5 respectively). L1 is a very severe case; the extension of the lesion is quite large, involving both GM and WM and presenting PH1 hemorrhage. L2 is less extensive compared to L1, although it too involves both GM and WM. Patients with worse outcomes present more heterogeneous lesions, often involving both GM and WM. In the presence of hemorrhage, heterogeneity in terms of radiomic features is even higher (e.g., texture heterogeneity measured in terms of Dependence non uniformity normalized—GLDM on DWI was 0.23 and 0.13 in L1 and L2, respectively). On the other hand, in patients that achieved functional independence (e.g., L3 and L4 presented a 90-day mRS of 2 and 0, respectively), the lesions were less heterogeneous (texture heterogeneity in terms of Dependence non uniformity normalized—GLDM on DWI of 0.08 and 0.04, respectively), and mostly involved GM areas.

## 4. Discussion

In this study, we showed the predictive value of radiomic features extracted from routinely acquired follow-up MR sequences (DWI and T2-FLAIR) for long-term functional outcome prediction in AIS patients. In the comparison of prediction models trained on five different feature sets, respectively including only radiomic features extracted by one follow-up MR sequence (DWI or T2-FLAIR), all the available imaging sequences (DWI and T2-FLAIR), only clinical characteristics, and finally a combination of all the radiomic features and the available clinical characteristics, we showed that the highest accuracy is found with the model trained on this last feature set. A completely data-driven ranking of the best model’s predictors based on SHAP values showed the relevance of radiomic features from both imaging modalities and confirmed, in line with previous clinical studies, the high predictive value of 24-h NIH Stroke Scale, and age [29,30].

Among the most relevant imaging characteristics is the heterogeneity of the lesion measured in terms of different texture features on both MR sequences, which has been shown to be more related to worse long-term functional outcome. This finding is interesting from a clinical perspective because heterogeneous lesions could indicate edema formation in the subacute stage or hemorrhage. In fact, focal cerebral ischemia and post-ischemic reperfusion can cause cerebral capillary dysfunction, resulting in edema formation and hemorrhagic conversion [31]. Because edema strongly influences patient prognosis, predicting its occurrence in the early stages is vital to providing timely intervention. However, there is a lack of early bio-markers, and the underlying physiological mechanisms of cerebral edema and hemorrhagic transformations are still being searched [32]. The work of Jiang et al. [33] supports our observation, showing how radiomic features from infarction and CSF (measured on DWI and T2-FLAIR respectively) may offer effective imaging bio-markers for predicting edema. In addition, Zhai et al. [34] showed DWI-based texture analysis with good predictive validity for hemorrhagic transformation in patients with acute massive cerebral infarction.

Another interesting aspect that can be captured by texture features is the involvement of white matter (WM) and gray matter (GM) in the ischemic lesion. Both GM and WM can be affected by ischemic stroke, as they need a constant supply of oxygen and glucose. However, the collateral circulation in WM is reduced compared to GM and the reduced blood supply makes WM highly vulnerable to ischemia, leading to more rapid and severe injuries [35]. Damage in WM integrity has been shown to be related to cognitive deficits in stroke patients and worse post-stroke functional outcomes [36]. More heterogeneous lesions are more likely to involve both GM and WM, indicating more severe injuries with fewer expectations of full functional recovery. Nonetheless, the lesions in patients with a favorable functional outcome were often smaller than those that did not achieve functional independence. Very large lesions are more likely to involve both GM and WM, resulting in a more heterogeneous areas, while very small lesions are often localized in GM or WM only. Therefore, heterogeneity indirectly takes into account follow-up lesion volume, which is known to be a useful prognostic factor [37]. Despite this, when the size of the lesion is comparable (e.g., L2, L3 in Figure 8), the involvement of both GM and WM still results in higher values of heterogeneity, indicating a higher risk of poor long-term functional outcome.

At present, most of the available radiomic evidence regarding stroke is derived from single-center studies, often leading to unstable and unreproducible association of radiomic features with clinical events due to selection bias [16]. This multi-center study suggests that functional outcome prediction using a combination of imaging and clinical characteristics leads to accurate results taking into account all the parameters routinely available up to 24 h. Moreover, we show that lesion heterogeneity measured from DWI and T2-FLAIR in terms of radiomic features can serve as a biomarker for poor clinical outcome. An accurate prognosis in the subacute stage is highly relevant for patients who ultimately have a poor outcome, as it enables early planning of care adapted to their needs [38]. Radiomics potential in stroke research has not been fully exploited yet, mostly due to the difficulty of collecting multi-center and large-scale imaging data. Nonetheless, the identification of stable imaging biomarkers would help to better understand the complex pathophysiological cascade involved in lesion formation and evolution after an AIS, optimize secondary prevention strategies, and facilitate the development of personalized precision medicine in post-stroke patients [16].

This study has a few limitations. Our results are not directly generalizable to patients not compliant to the MR CLEAN-NO IV inclusion criteria. Therefore, future studies assessing the impact of the identified predictors for stroke patients with minor vessel occlusions or posterior circulation occlusions are needed. Moreover, we showed that 24-h radiomic features, if used on top of clinical characteristics, have the potential to improve long-term prognosis prediction. However, in our study the differences are small. This is most likely due to the relatively small size of the analyzed sample. Future works including a larger population could probably lead to increased statistical significance and model performance. Furthermore, with a larger sample size it would be interesting to try end-to-end DL approaches comparing the predictive power and interpretability of radiomic and automatically extracted features. Research in this field is in its early stages; studies on larger populations are needed to further assess the scalability and robustness of the proposed method and the identified biomarkers, which is essential before considering its use in the clinical practice. Finally, we only considered mRS score as long-term functional outcome, as it is the most used indicator in clinical practice; however different scores better reflecting the psychological and emotional status of the patient could be used as endpoints, possibly leading to a better understanding of the disease mechanisms.

## 5. Conclusions

In this study, we showed that radiomic imaging features describing infarct lesion on follow-up MR sequences (DWI and T2-FLAIR) are predictive of poor long-term functional outcome in AIS patients, with features describing texture heterogeneity being the most predictive imaging biomarkers. In addition, we proved that using imaging features on top of routinely available clinical characteristics, and thus exploiting all patient data available up to 24 h, leads to the most accurate prognostic model. Further research in this direction may help in better targeting secondary treatments to mitigate lesion evolution in the subacute phase and thereby improving long-term functional outcome, hopefully reducing the burden of disease.

## Figures and Tables

**Table 1:** Average metrics (95% CI) computed on the test sets for all the tested feature sets (*DWI set*: only DWI radiomic features, *FLAIR set*: only T2-FLAIR radiomic features, *imaging set*: both DWI and T2-FLAIR radiomic features, *clinical set*: only clinical characteristics, *combination set*: combination of all the radiomic features and the clinical characteristics). For each feature set, Accuracy (Acc), Sensitivity (Sens), Specificity (Spec), Precision (Prec), and Area under the ROC curve (AUC) are reported.

Feature Set	Acc	Sens	Spec	Prec	AUC
DWI	0.66[0.56,0.76]	0.58[0.38,0.78]	0.72[0.56,0.88]	0.62[0.48,0.76]	0.69[0.55,0.82]
FLAIR	0.64[0.58,0.70]	0.56[0.46,0.66]	0.71[0.62,0.80]	0.58[0.50,0.66]	0.68[0.59,0.77]
Imaging	0.67[0.60,0.74]	0.56[0.47,0.65]	0.76[0.66,0.86]	0.65[0.55,0.75]	0.71[0.63,0.79]
Clinical	0.75[0.70,0.80]	0.71[0.58,0.84]	0.79[0.74,0.84]	0.72[0.67,0.77]	0.83[0.78,0.88]
Combination	0.79[0.72,0.86]	0.71[0.62,0.80]	0.84[0.74,0.94]	0.78[0.68,0.88]	0.85[0.81,0.89]

**Table 2:** DeLong’s test *p*-values for pairwise AUC comparisons (α=0.05), with the highest AUC as a reference. Statistically significant results are provided in bold.

Feature Set	AUC	*p*-Value
Combination—DWI	0.85–0.69	**<0.001**
Combination—FLAIR	0.85–0.68	**<0.001**
Combination—Imaging	0.85–0.71	**<0.001**
Combination—Clinical	0.85–0.83	0.33

## Data Availability

Trial data can be made available on reasonable request via mrclean@erasmusmc.nl.

## Supplementary Materials

The following supporting information can be downloaded at: https://www.mdpi.com/article/10.3390/jcdd9120468/s1, Table S1: Comparison of baseline clinical/imaging characteristics, treatment and post-treatment clinical characteristics between patients with favorable (90-day mRS ≤ 2) and unfavorable (90-day mRS > 2) long-term clinical outcome; Table S2: Comparison of baseline clinical/imaging characteristics, treatment and post-treatment clinical characteristics between included and excluded patients from MR CLEAN-NO IV trial.
