# Supplementary material for: Prognostic Value of Combined Radiomic Features from Follow-Up DWI and T2-FLAIR in Acute Ischemic Stroke
Source: J Cardiovasc Dev Dis. 2022 Dec 19;9(12):468. doi: 10.3390/jcdd9120468 (PMC9786822; doi:10.3390/jcdd9120468)
Supplement: Supplementary file 1 [file jcdd-09-00468-s001.zip › jcdd-2073842-supplementary.pdf]

# Supplementary Materials:

**Table S1.** Comparison of baseline clinical/imaging characteristics, treatment and post-treatment clinical characteristics between patients with favorable (90-day mRS$\leq$2) and unfavorable (90-day mRS>2) long-term clinical outcome. For each feature number(%population) or median(IQ1-IQ3) are reported. Missing values are provided as number(%population). Mann-Whitney U test and Fisher/Chi-Square tests ($\alpha = 0.05$) were performed to compare continuous and binary/categorical variables respectively between patients with different clinical outcome.

| **Characteristic** | | **Favorable functional outcome (90-day mRS≤2) n = 93** | **Unfavorable functional outcome (90-day mRS>2) n = 71** | **p-value** |
|---|---|---|---|---|
| Age | | 68 (61-76) | 73 (66-83) | <0.01 |
| Male sex | | 56 (60.2%) | 35 (49.3%) | 0.20 |
| **Baseline clinical and imaging characteristics** | | | | |
| Previous ischemic stroke | | 12 (12.9%) | 11 (15.5%) | 0.66 |
| Atrial fibrillation | | 2 (2.2%) | 9 (12.7%) | 0.01 |
| Diabetes Mellitus | | 7 (7.5%) | 10 (14.1%) | 0.20 |
| Hypertension | | 42 (45.2%) | 36 (50.7%) | 0.53 |
| Glucose | | 6.3 (5.4-7.4) | 6.8 (5.9-8.2) | 0.02 |
| Systolic Blood Pressure | | 145.0 (135.0-160.0) | 150.0 (130.0-165.5) | 0.30 |
| Baseline NIH Stroke Scale | | 15.0 (9.0-19.0) | 17.0 (11.0-21.0) | 0.01 |
| ASPECTS | | 9.0 (8.0-10.0) | 9.0 (9.0-10.0) | 0.24 |
| Collateral score | Absent | 2 (2.2%) | 8 (11.3%) | 0.01 |
| | <50% | 17 (18.3%) | 24 (33.8%) | |
| | 50-100% | 44 (47.3%) | 24 (33.8%) | |
| | 100% | 27 (29.0%) | 14 (19.7%) | |
| | Missing | 3 (3.2%) | 1 (1.4%) | |
| Ipsilateral extracranial carotid tandem lesion | n (%) | 18 (19.4%) | 15 (21.1%) | 0.84 |
| | Missing | 4 (4.3%) | 4 (5.6%) | |
| Right hemisphere stroke | | 42 (45.2%) | 32 (45.1%) | 1.00 |
| Occlusion location | M1 | 56 (60.2%) | 44 (62.0%) | 0.77 |
| | M2 | 15 (16.1%) | 9 (12.7%) | |
| | ICA | 22 (23.7%) | 17 (23.9%) | |
| | Other | 0 (0.0%) | 1 (1.4%) | |
| Pre-stroke modified Rankin Scale | 0 | 83 (89.2%) | 45 (63.4%) | <0.01 |
| | 1 | 9 (9.7%) | 17 (23.9%) | |
| | 2 | 1 (1.1%) | 9 (12.7%) | |
| | >3 | 0 (0.0%) | 0 (0.0%) | |
| **Treatment and post-treatment characteristics** | | | | |
| Treatment allocation (EVT alone) | | 53 (57.0%) | 35 (49.3%) | 0.35 |
| Onset to randomisation (minutes) | | 104.0 (64.0-145.0) | 93.0 (68.0-151.5) | 0.45 |
| Onset to needle (minutes) | Median (IQR) | 104.5 (64.0-158.5) | 95.0 (76.0-160.0) | 0.32 |
| | Missing | 53 (57.0%) | 36 (50.7%) | |
| Door to groin (minutes) | Median (IQR) | 63.0 (49.8-78.0) | 59.0 (49.0-69.5) | 0.13 |
| | Missing | 1 (1.1%) | | |
| Door to needle (minutes) | Median (IQR) | 28.0 (17.8-48.0) | 29.0 (21.5-40.5) | 0.27 |
| | Missing | 53 (57.0%) | 36 (50.7%) | |
| Onset to groin (minutes) | Median (IQR) | 133.0 (99.0-176.3) | 130.0 (105.5-180.5) | 0.49 |
| | Missing | 1 (1.1%) | | |
| Needle to groin (minutes) | Median (IQR) | 23.0 (16.5-37.0) | 28.0 (18.5-38.5) | 0.26 |
| | Missing | 54 (58.1%) | 36 (50.7%) | |
| 24-hour NIH Stroke Score | | 3.0 (1.0-5.0) | 12.0 (7.0-16.0) | <0.01 |

**Table S2.** Comparison of baseline clinical/imaging characteristics, treatment and post-treatment clinical characteristics between included and excluded patients from MR CLEAN-NO IV trial. For each feature number(%population) or median(IQ1-IQ3) are reported. Missing values are provided as number(%population). Mann-Whitney U test and Fisher/Chi-Square tests were performed to compare continuous and binary/categorical variables respectively between included and excluded patients. This table also shows the statistics of the same characteristics for the whole MR CLEAN-NO IV population to indicate potential differences in our sub-population. Although there are few statistically significant differences between some clinical characteristics when comparing the sample and the original population, none of these was selected among the best predictive features by the TreeSHAP algorithm.

| **Characteristic** | | **NO-IV population (n = 539)** | **Included in study population** | | **p-value** |
|---|---|---|---|---|---|
| | | | **No (n = 375)** | **Yes (n =164)** | |
| Age | | 71(62-79) | 71(61-79) | 71(63-78) | 0.93 |
| Male sex | | 305(57%) | 214(57%) | 91(55%) | 0.78 |
| **Baseline clinical and imaging characteristics** | | | | | |
| Previous ischemic stroke | | 91(17%) | 68(18%) | 23(14%) | 0.26 |
| Atrial fibrillation | | 58(11%) | 47(13%) | 11(7%) | 0.05 |
| Diabetes mellitus | | 90(17%) | 73(19%) | 17(10%) | 0.01 |
| Hypertension | n (%) | 260(48%) | 182(49%) | 78(48%) | 0.85 |
| | Missing | 1 (0.19%) | 1(0.27%) | 0 | |
| Glucose | Median (IQR) | 6.7(5.9-7.9) | 6.8(6.0-8.0) | 6.0(5.6-7.6) | 0.02 |
| | Missing | 7(1.30%) | 7(1.87%) | 0 | |
| Systolic blood pressure | Median (IQR) | 150(130-170) | 150(134-170) | 149(131-163) | 0.14 |
| | Missing | 2(0.37%) | 2(0.53%) | 0 | |
| Baseline NIH Stroke Scale | | 16(10-20) | 16(10-20) | 15(10-20) | 0.61 |
| ASPECTS | | 9(8-10) | 9(8-10) | 9(8-10) | 0.10 |
| Collateral score | Absent | 32(6%) | 22(6%) | 10(6%) | 0.62 |
| | <50% | 152(28%) | 111(30%) | 41(25%) | |
| | 50-100% | 223(41%) | 155(41%) | 68(41%) | |
| | 100% | 119(22%) | 78(21%) | 41(25%) | |
| | Missing | 13(2.4%) | 9(2.4%) | 4(2.4%) | |
| Ipsilateral extracranial carotid tandem lesion | n (%) | 88(16%) | 55(15%) | 33(20%) | 0.16 |
| | Missing | 32(5.9%) | 24(6.4%) | 8(5%) | |
| Right hemisphere stroke | | 249(46%) | 175(47%) | 74(45%) | 0.78 |
| Occlusion location | M1 | 330(61%) | 230(61%) | 100(61%) | 0.37 |
| | M2 | 85(16%) | 61(16%) | 24(15%) | |
| | ICA | 118(22%) | 79(21%) | 39(24%) | |
| | Other | 5(1%) | 4(1%) | 1(0.6%) | |
| | Missing | 1(0.19%) | 1(0.26%) | 0 | |
| Pre-stroke modified Rankin Scale | 0 | 374(69%) | 246(66%) | 128(78%) | 0.01 |
| | 1 | 100(19%) | 74(20%) | 26(16%) | |
| | 2 | 49(9%) | 39(10%) | 10(6%) | |
| | 3 | 11(2%) | 11(3%) | 0 | |
| | 4 | 4(1%) | 4(1%) | 0 | |
| | 5 | 0 | 0 | 0 | |
| | Missing | 1(0.19%) | 1(0.26%) | 0 | |
| **Treatment and post-treatment characteristics** | | | | | |
| Treatment allocation (EVT alone) | | 273(51%) | 185(49%) | 88(54%) | 0.40 |
| Onset to randomization (minutes) | | 93(71-145) | 92(71-143) | 96(65-150) | 0.87 |
| Onset to needle (minutes) | Median (IQR) | 100(75-156) | 100(80-150) | 95(66-159) | 0.22 |
| | Missing | 279(52%) | 190(51%) | 89(54%) | |
| Door to groin (minutes) | Median (IQR) | 64(51-78) | 65(52-79) | 60(49-73) | 0.02 |
| | Missing | 28(5%) | 27(7%) | 1(0.6%) | |
| Door to needle (minutes) | Median (IQR) | 31(24-44) | 33(25-44) | 29(19-45) | 0.12 |
| | Missing | 279(52%) | 190(51%) | 89(54%) | |
| Onset to groin (minutes) | Median (IQR) | 133(105-180) | 135(105-180) | 130(103-180) | 0.31 |
| | Missing | 28(5%) | 27(7%) | 1(0.6%) | |
| Needle to groin (minutes) | Median (IQR) | 28(20-41) | 29(20-43) | 25(17-39) | 0.13 |
| | Missing | 298(55%) | 208(55%) | 90(55%) | |
| 24-hours NIH Stroke Score | | 6(1-14) | 6(2-15) | 15(10-20) | 0.15 |
| **Unfavorable functional outcome (90-day mRS>2)** | | 269(50%) | 198(53%) | 71(43%) | 0.06 |
